# ADVANCES IN INTERDISCIPLINARY SCIENCE: BRAIN CONTROL OF MOBILITY IN AGING AND DISEASE POPULATIONS

**DOI:** 10.1093/geroni/igae098.0146

**Published:** 2024-12-31

**Authors:** Roee Holtzer, Teresa Liu-Ambrose

**Affiliations:** Chair; Discussant

## Abstract

Interdisciplinary research has demonstrated that cognitive and mobility performance and decline are inter-related and potentially coupled implicating the brain as a key common denominator shared by both domains of function. Novel studies in the current symposium utilize advanced neuroimaging methods to elucidate how structural and functional measures of brain integrity are utilized to predict mobility outcomes and mitigate against risk of developing mobility decline and impairments. Mark Wagshul demonstrates that whole brain latent patterns have differential associations with gait speed in older adults with and without multiple sclerosis. Paul Laurienti uses brain networks connectivity data to define cognitive reserve demonstrating that associations between amyloid beta and mobility decline are attenuated among older adults with high cognitive reserve. Chun Liang Hsu uses functional neural networks to define a novel construct - physical reserve. He demonstrates that among older adults with subcortical ischemic vascular cognitive impairment (SIVCI), high physical reserve attenuates the negative effect of white matter hyperintensities on postural stability. Roee Holtzer uses functional-near-infrared-spectroscopy (fNIRS) to assess oxygenated hemoglobin (HbO) in the prefrontal cortex during walking under single and dual-task conditions. He shows that higher fNIRS-derived HbO during dual-task walking is protective against future falls in older adults with relapsing-remitting but not progressive MS subtype. Combined, these studies shed light on novel discoveries concerning brain control of mobility in aging and disease populations. Furthermore, emphasis is placed on demonstrating how brain data are utilized to define constructs (cognitive and physical reserve) and elucidate theoretical models (compensatory reallocation) vis-à-vis mobility outcomes. Brain Interest Group Sponsored Symposium

